# Effect of leisure-time physical activity on depression and depressive symptoms in menopausal women: a systematic review and meta-analysis of randomized controlled trials

**DOI:** 10.3389/fpsyt.2024.1480623

**Published:** 2025-01-30

**Authors:** Rong Liu, Xun Tang

**Affiliations:** College of Physical Education, Chengdu University, Chengdu, China

**Keywords:** physical activity, depressive symptoms, depression, menopausal women, depression degree

## Abstract

**Aims:**

Menopausal women often suffer from depression, which impairs their quality of life. Physical activity has been reported to exert beneficial effects on preventing and treating depression. This meta-analysis aims to explore the effect of leisure-time physical activity on determined depression or depressive symptoms in menopausal women.

**Methods:**

Relevant studies were searched from PubMed, Embase, Cochrane Library, Web of Science, PsycINFO, CINAHL Plus, China National Knowledge Infrastructure (CNKI), VIP, and WanFang databases. Outcomes were depression or depressive symptoms. Weighted mean difference (WMD) or standard mean difference (SMD) with 95% confidence interval (CI) was used as the statistical measure. Heterogeneity tests were performed for each outcome, and all outcomes were subjected to sensitivity analysis. Subgroup analysis was performed based on depression degree, exercise intensity, exercise form, intervention duration, supervision, sample size, and geographical region.

**Results:**

A total of 17 studies were included in this meta-analysis. The results showed that exercise alleviated the depressive symptoms of menopausal women (SMD = −1.23; 95% CI, −2.21 to −0.24). In addition, exercise was found to reduce the depression (SMD = 11.45; 95% CI, −1.75 to −1.15), and depression assessed by the Center for Epidemiologic Studies Depression Scale (CES-D) (WMD = −5.76; 95% CI, −6.63 to −4.89) or Self-Rating Depression Scale (SDS) (WMD = −6.86; 95% CI, −9.24 to −4.49). The results were similar regardless of depression degrees, exercise intensity, intervention duration, exercise form, supervision or not, sample size, and geographical region.

**Conclusions:**

Leisure-time physical activity may help alleviate depressive symptoms or depression in menopausal women. However, further high-quality studies are needed to confirm these findings and better understand the specific effects of physical activity on depression in this population.

**Systematic review registration:**

https://www.crd.york.ac.uk/prospero/, identifier CRD42024581087.

## Introduction

Menopause refers to the permanent cessation of ovarian function, and menstruation is stopped for at least 12 months (1). Menopausal women will experience hot flushes, night sweats, vaginal dryness, mood swings, insomnia, and depression (2). The global prevalence of depression in menopausal women was 35.6%, with 33.9% in perimenopausal women and 34.9% in postmenopausal women (3). Depression in menopausal women has the potential to impair functional outcomes, reduce quality of life, and decrease satisfaction of life; therefore, it is important to improve depression or depressive symptoms in menopausal women (4).

Although depression is typically treated with medication and psychotherapy in clinical settings, interventions based on physical activities are increasingly recognized as an affordable, non-invasive, and easily accessible treatment for depression (5–7). Exercise has been linked to increased blood flow to the brain (8) and neurotransmitter levels, enhanced better focus (9), and improved sleep quality (10). The beneficial effect of physical activity on preventing and treating depression was observed (11). Schuch et al. reported that people with high levels of physical activity had 17% lower odds of developing depression than those with low levels (12). Brinsley et al. found that physical activity showed greater reductions in depressive symptoms than usual treatment, and greater improvement was associated with higher frequency (13). In menopausal women, the beneficial roles of physical activity in depression are also reported (14, 15). Lialy et al. found that yoga and walking decreased depression scores and the incidence of depression in menopausal women (14). Aibar-Almazán et al. performed a randomized controlled trial (RCT) in 110 community-dwelling Spanish postmenopausal women, and results showed that a 12-week Pilates exercise intervention contributed to reducing the depression score (15). A previous meta-analysis had reported that exercise of low to moderate intensity could alleviate depressive symptoms in midlife and older women (16); however, this meta-analysis mixed women with determined depression and depressive symptoms and also does not include patients with severe depression.

In this meta-analysis, we explored the effect of physical activity on menopausal women with determined depression or depressive symptoms, respectively. We also performed subgroup analysis based on depression degrees.

## Methods

The standard Cochrane methods were used in this meta-analysis, which were performed according to Preferred Reporting Items for Systemic Reviews and Meta-Analyses (PRISMA) guidelines (17). We have registered our review in PROSPERO, and the registration number is CRD42024581087.

### Literature search strategy

A thorough literature review was conducted by searching multiple databases including PubMed, Embase, Cochrane Library, Web of Science, PsycINFO, CINAHL Plus, China National Knowledge Infrastructure (CNKI), VIP, and WanFang. The search spanned from the inception of each database up to 13 November 2024. Database searches were performed by two independent authors according to the following terms: “Aerobic” OR “Jog” OR “Walk” OR “Pilates” OR “Strength training” OR “Stretching” OR “Ambulation” OR “Yoga” OR “Swim” OR “Dance” OR “Dancing” OR “strengthening” OR “warming up” OR “cooling down” OR “Exercise” OR “Exercises” OR “Physical Activity” OR “Activities, Physical” OR “Activity, Physical” OR “Physical Activities” OR “Exercise, Physical” OR “Exercises, Physical” OR “Physical Exercise” OR “Physical Exercises” OR “Acute Exercise” OR “Acute Exercises” OR “Exercise, Acute” OR “Exercises, Acute” OR “Exercise, Isometric” OR “Exercises, Isometric” OR “Isometric Exercises” OR “Isometric Exercise” OR “Exercise, Aerobic” OR “Aerobic Exercise” OR “Aerobic Exercises” OR “Exercises, Aerobic” OR “Exercise Training” OR “Exercise Trainings” OR “Training, Exercise” OR “Trainings, Exercise” AND “perimenopause” OR “menopause” OR “menopausal” OR “postmenopause” OR “postmenopausal” OR “climacteric” OR “perimenopausal” AND “Depression” OR “Depressive Symptoms” OR “Depressive Symptom” OR “Symptom, Depressive” OR “Emotional Depression” OR “Depression, Emotional”. The search strategy of PubMed database is shown in **Supplementary Data Sheet 1**.

### Inclusion and exclusion criteria

Inclusion criteria were as follows: (1) population—menopausal women; (2) intervention—physical activities; (3) control—daily activities or other therapies not involving physical activities, such as dietary restrictions and psychological counseling; (4) outcome—depression or depressive symptoms assessed by Beck Depression Inventory (BDI), Self-Rating Depression Scale (SDS), Center for Epidemiologic Studies Depression Scale (CES-D), Geriatric Depression Scale (GDS) or Depression Anxiety Stress Scales-21 Items (DASS-21); and (5) study type—RCTs.

Exercise intensity was classified according to the original literature. For literature not reporting the exercise intensity, the exercise intensity was classified according to the 2011 Compendium, and metabolic equivalent (MET) values were recorded for each activity (18). Physical activity was divided into sedentary behavior (1.0–1.5 METs), light intensity (1.6–2.9 METs), moderate intensity (3–5.9 METs), and vigorous intensity (≥6 METs) (18). MET/week = exercise duration (min) × MET × frequency/week.

Exclusion criteria were as follows: (1) animal experiments; (2) studies unable to extract valid data; (3) conference abstracts, case reports, meta-analyses, reviews, editorial materials, letters, trial registry records, or guidelines; (4) not published in Chinese or English; and (5) retracted articles.

### Data extraction

Two independent authors extracted the following data: the first author, publication year, country, study design, sample size, population/depression degree, age, intervention, exercise intensity, METs, frequency, MET/week, intervention duration, exercise form (individual or team), supervised, comparison group, and depression assessment tool. A third researcher was consulted if disagreements appeared.

### Quality assessment

The quality of RCTs was assessed using the Cochrane risk of bias tool, which assessed the included studies based on seven items (generation of random sequence, allocation concealment, blinding of participants and personnel, blinding of outcome assessment, incomplete outcome data, selective reporting bias, and other bias) (19). Each domain was divided into three categories: “low risk,” “high risk,” and “unclear risk” (19).

In this study, we used the Grading of Recommendations, Assessment, Development, and Evaluations (GRADE) system to assess the quality of evidence. The GRADE system evaluates the strength of evidence based on factors such as study design, risk of bias, inconsistency, indirectness, imprecision, and publication bias (**Supplementary Data Sheet 2**).

### Statistical analysis

When physical activity was considered as a treatment, the weighted mean difference (WMD) was utilized to compare outcomes between the intervention group and the control group. Given the variability in scales assessing, this study also applied the standardized mean difference (SMD) to compare outcomes between the intervention group and the control group. In our analysis of physical activity as a preventive measure against depressive symptoms, SMD was employed as the effect index. The effect size was expressed as a 95% confidence interval (CI). Heterogeneity tests were conducted on the effect size of each outcome. In meta-analysis, assessing heterogeneity is crucial because it quantifies the variability in study outcomes that is not due to chance. The I^2^ statistic is a measure of this heterogeneity, and it provides an estimate of the percentage of the variability in effect sizes that is due to heterogeneity rather than sampling error. When the I^2^ value is 0%, it suggests no observed heterogeneity, and larger values indicate increasing levels of heterogeneity. If the heterogeneity statistic I^2^ ≥ 50%, the random-effects model was used; otherwise, the fixed effects model was used. Subgroup analysis was performed based on depression degree (mild depression and mild to moderate depression), exercise intensity (low, moderate, and moderate–vigorous), exercise form (individual exercise and team exercise), intervention duration (≤12 weeks and >12 weeks), supervised (yes or no), sample size, and geographical region. Meta-regression used in the meta-analysis was to explore whether specific study-level variables (also known as covariates or moderators) can explain variability (heterogeneity) in effect sizes across studies. All outcomes underwent sensitivity analysis to assess the robustness and stability of the results. This involved systematically re-evaluating the data by excluding individual studies one at a time to observe any changes in the overall effect size and confidence interval. By identifying how each study impacted the pooled results, the sensitivity analysis helped determine whether the findings were influenced by any single study or were consistent across the included studies. Publication bias was not assessed due to the limited number of studies included for each outcome, with fewer than 10 studies available. As tests for publication bias, such as funnel plots or Egger’s test, require a minimum of 10 studies to produce reliable results, conducting these tests with fewer studies could lead to inaccurate or misleading conclusions (20). RevMan 5.3 (Cochrane Collaboration, Oxford, UK) was used to evaluate the quality of RCTs and generate the risk of bias graph and risk of bias summary. Statistical analysis for all studies was conducted using Stata15.1 software (StataCorp, College Station, TX, USA).

## Results

### Study characteristics and quality assessment

**Figure 1:**
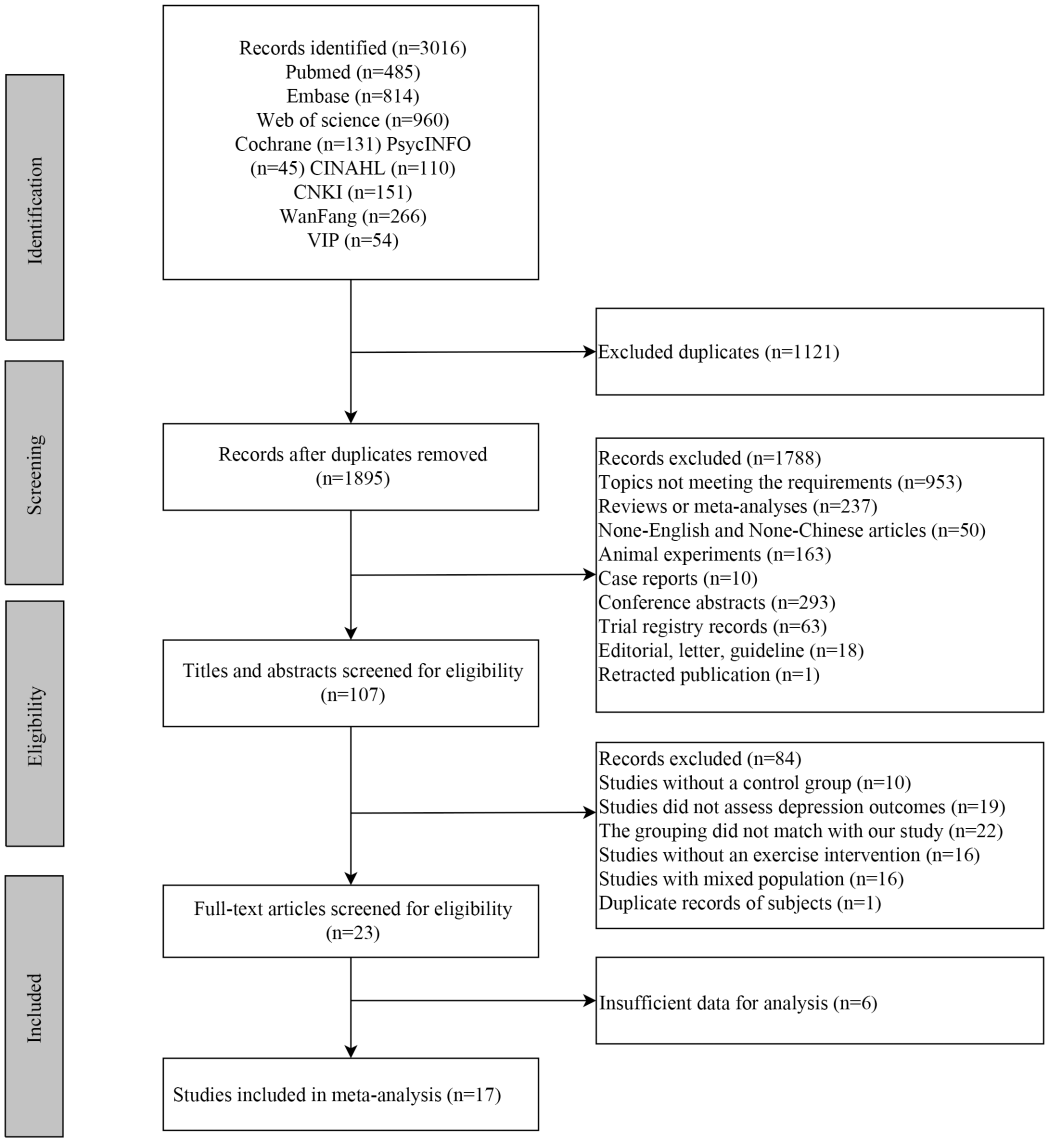
The flowchart of studies selection.

A total of 3,016 publications were identified from the above-mentioned databases. After excluding 1,121 duplicates, 1,895 publications remained. After screening titles and abstracts, 1,788 publications were excluded. In the remaining 107 publications, 84 publications were further excluded due to subjects not meeting the requirements after screening the full text (**Supplementary Data Sheet 3**). Finally, 17 eligible studies were included in this meta-analysis (1, 21–36) (**Figure 1**).

**Figure 2:**
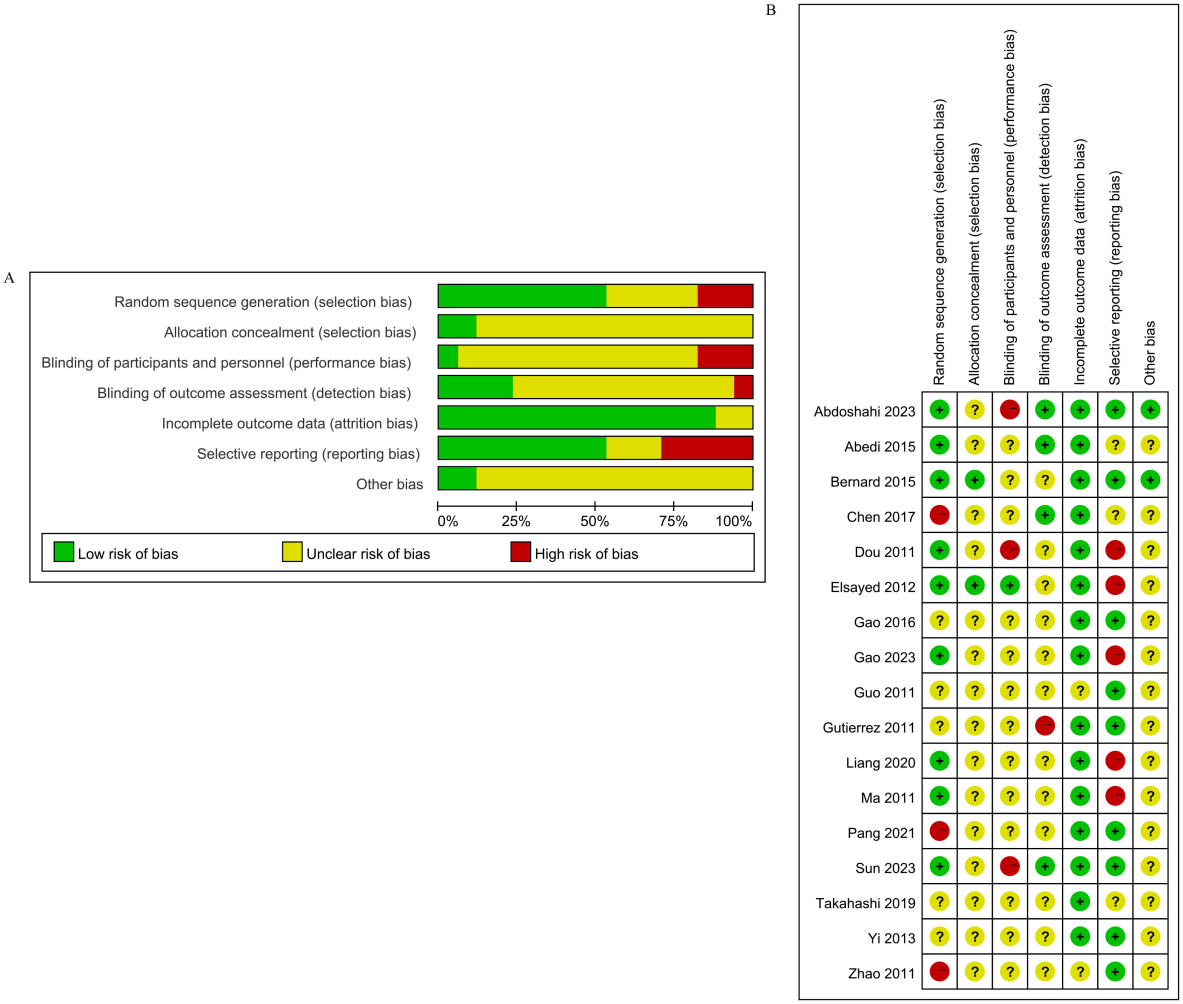
Risk of bias graph **(A)** and summary **(B)** for the included RCTs. RCT, randomized controlled trial.

Of the included studies, six studies (21, 31, 32, 34–36) reported the depressive symptoms in menopausal women. The characteristics of these studies are shown in **Table 1**. A total of 11 studies (1, 22–30, 33) reported depression in menopausal women, and the characteristics are shown in **Table 2**. Risk of bias assessment is shown in **Figures 2A, B**.

**Table 1:** Characteristics of studies including menopausal women with depressive symptoms.

Author	Year	Country	Study design	Sample size (EX; CG)	Population	Age (EX; CG)	Intervention	Intensity of exercise	METs	Frequency (weekly)	MET/week	Duration of intervention	Exercise form (individual or team)	Supervised	Comparison group	Depression assessment tool
Bernard	2015	France	RCT	61;60	Postmenopausal	65.46 ± 4.37; 65.5 ± 4.03	Walking	moderate	5.3	3	636	6 months	IE	yes	Usual lifestyle	BDI
Takahashi	2019	Japan	RCT	19;19	Postmenopausal	70.2 ± 3.9	Increase physical activities	NR	NR	NR	NR	8weeks	IE	No	Usual lifestyle	GDS
Zhao	2011	China	RCT	64;64	Menopausal	47.12 ± 3.25; 47.31 ± 3.61	Yoga	Low	2.5	2	300	16 weeks	TE	yes	Usual lifestyle	CES-D
Pang	2021	Korea	RCT	18;12	Postmenopausal	60.89 ± 6.62; 59.42 ± 5.16	Calendar training + walking	moderate	3.5	7	1470	12 weeks	IE	yes	Calendar training	GDS
Abdoshahi	2023	Iran	RCT	16;16	Menopausal	EX: 50-55, 9; 56-60, 6; 61-65, 1; CG: 50-55, 10; 56-60,4; 61-65, 2	Pilates exercises	moderate	3	2	NR	12 weeks	TE	yes	Regular daily activities	DASS-21
Sun	2023	China	RCT	32;32	Peri-menopausal	51.28 ± 3.05; 50.65 ± 2.76	Aerobic exercise combined with resistance exercise	NR	NR	Aerobic exercise at least 3 times, resistance exercise 2 times	NR	6 months	IE	No	Usual lifestyle	SDS

EX, exercise; CG, control group; MET, metabolic equivalent; IE, individual exercise; TE, team exercise; BDI, Beck Depression Inventory; SDS, Self-Rating Depression Scale; GDS, Geriatric Depression Scale; CES-D, Center for Epidemiologic Studies Depression Scale.

**Table 2:** Characteristics of studies including menopausal women with depression.

Author	Year	Country	Study design	Sample size (EX; CG)	Depression degree	Age (EX; CG)	Intervention	Exercise intensity	METs	Frequency (weekly)	MET/week	Intervention duration	Exercise form (individual or team)	Supervised	Comparison group	Depression assessment tool
Abedi	2015	Iran	RCT	49;48	mild to moderate depression	52.4 ± 3.8; 53 ± 4.1	pedometer-based walking	low	2	increase≥500 steps/week	NR	12 weeks	IE	No	no intervention	BDI
Gao	2016	China	RCT	26;24	mild to severe depression	54.5 ± 4.5; 53.5 ± 4.7	square dance	vigorous	7.8	5	1350	3 months	TE	yes	usual lifestyle	SDS
Gutierrez	2011	Spain	RCT	30;30	moderate to severe depression	63.5 ± 3.3; 64.2 ± 2.8	aerobic exercise + muscle training exercises	moderate	3.8	2	380	6 months	TE	yes	usual lifestyle	GDS
Chen	2017	China	RCT	jog: 25, resistance exercise: 24, qigong: 27; CG:24	mild depression	(45,55)^	jog; resistance; qigong	jog, moderate; resistance exercise, moderate; qigong, moderate	jog, 4.5; resistance exercise, 3.5; qigong, 3.0	3-4	jog, 405; resistance exercise, 472.5; qigong, 270	24 weeks	TE	yes	usual lifestyle	SDS
Gao	2023	China	RCT	40;40	mild to moderate depression	54.75 ± 4.84; 53.1 ± 5.24	routine follow-up + baduan jin exercise	moderate	3	NR	NR	12 weeks	IE	No	usual lifestyle	SDS
Guo	2011	China	RCT	30;30	moderate to severe depression	48.1 ± 2.8; 46.3 ± 2.6	skipping rope exercise	vigorous	8.8	3	1188	3 months	NR	NR	usual lifestyle	CES-D
Liang	2020	China	RCT	48;48	moderate to severe depression	49.28 ± 1.13; 49.32 ± 1.07	skipping rope exercise	vigorous	8.8	3	NR	3 months	NR	NR	usual lifestyle	CES-D
Ma	2011	China	RCT	walking:46, baduan jin exercise:49; CG:50	moderate to severe depression	walking:47.68 ± 2.56, baduan jin exercise:46.89 ± 2.69; CG:46.92 ± 2.31	walking; baduan jin exercise	moderate	3	walking, 5; baduan jin exercise, NR	walking, 675; baduan jin exercise, NR	3 months	TE	yes	usual lifestyle	CES-D
Dou	2011	China	RCT	skipping rope exercise:50, baduan jin exercise:50; CG:49	moderate to severe depression	skipping rope exercise:48.1 ± 2.8, baduan jin exercise:46.3 ± 2.6; CG:46.5 ± 2.1	skipping rope exercise; baduan jin exercise	skipping rope exercise, vigorous; baduan jin exercise, moderate	skipping rope exercise, 8.8; baduan jin exercise, 3	skipping rope exercise, 3; baduan jin exercise, 5	skipping rope exercise, 1,188; baduan jin exercise, 675	20weeks	TE	yes	usual lifestyle	CES-D
Elsayed	2022	Egypt	RCT	30;30	mild depression	58.99 ± 3.22; 58.58 ± 2.37	jog + balanced-restricted diet	low-moderate	5.9	3	885	12 weeks	IE	yes	balanced-restricted diet	SDS
Yi	2013	China	RCT	50;50	mild to moderate depression	49.72 ± 2.42; 49.86 ± 3.19	dance	moderate	4.5	5	900	24 weeks	TE	yes	usual lifestyle	SDS

^, (min,max); EX, exercise; CG, control group; MET, metabolic equivalent; IE, individual exercise; TE, team exercise; BDI, Beck Depression Inventory; SDS, Self-Rating Depression Scale; GDS, Geriatric Depression Scale; CES-D, Center for Epidemiologic Studies Depression Scale.

### Effect of physical activity on the prevention of depressive symptoms in menopausal women

**Figure 3:**
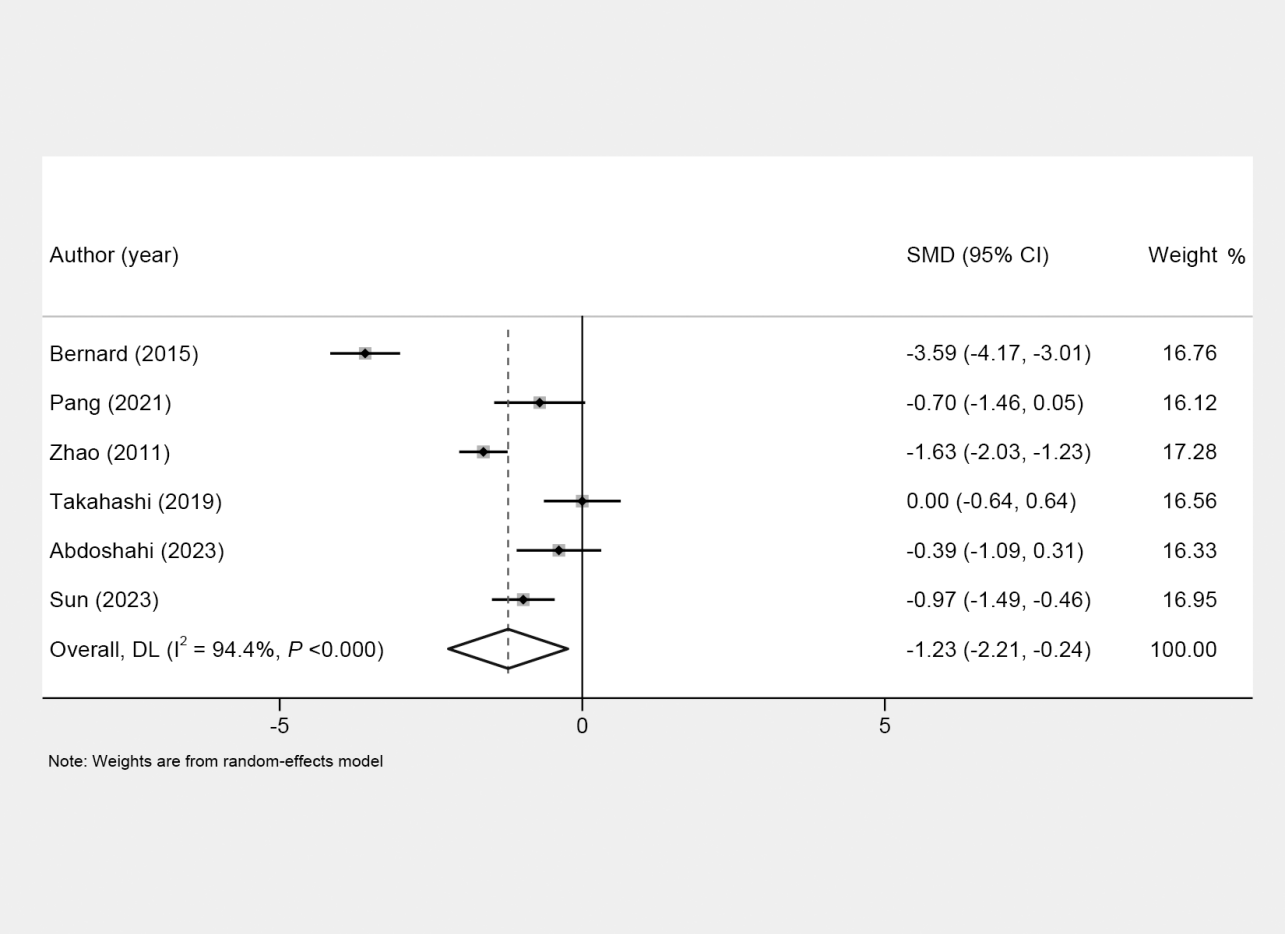
Forest plots of the effects of physical activity on depressive symptoms of menopausal women.

After exercise, depressive symptoms of menopausal women were alleviated in the intervention group compared to the control group (SMD = −1.23; 95% CI, −2.21 to −0.24; I^2^ = 94.4%) (**Figure 3**, **Table 3**). As for exercise intensity, depressive symptoms were reduced in menopausal women receiving low-intensity exercise (SMD = −1.63; 95% CI, −2.03 to −1.23). As for intervention duration, exercise >12 weeks was found to improve depressive symptoms (SMD = −2.60; 95% CI, −4.51 to −0.68). In addition, the depressive symptoms were alleviated after exercise under supervision (SMD = −1.59; 95% CI, −2.91 to −0.27) (**Table 3**). Physical activity was found to significantly reduce depressive symptoms in menopausal women as measured by the BDI (SMD = −3.58; 95% CI, −4.17 to −3.01), CES-D (SMD = −1.63; 95% CI, −2.03 to −1.23; *p <*0.001), and SDS (SMD = −0.98; 95% CI, −1.49 to −0.46; *p <*0.001). However, no significant effects were observed using the GDS (SMD = −0.32; 95% CI, −1.01 to 0.37; *p* =0.359) or DASS-21 (SMD = −0.386; 95% CI, −1.086 to 0.313; *p* =0.279). In terms of sample size, studies with ≥100 participants (SMD = −2.60; 95% CI, -4.51 to −0.68; *p* =0.008) and <100 participants (SMD = −0.535; 95% CI, −0.983 to −0.087, *p* =0.019) both showed a significant reduction in depressive symptoms after exercise (**Table 3**). In terms of geographical region, a significant reduction in depressive symptoms was observed after exercise in studies conducted in Europe (SMD = −3.59; 95% CI, −4.17 to −3.01; *p <*0.001). In East Asia, the effect was moderate and statistically significant (SMD = −0.89; 95% CI, −1.57 to −0.15; *p* = 0.018). However, Middle Eastern studies did not show a significant effect (SMD = −0.39; 95% CI, −1.09 to 0.31; *p* =0.279) (**Table 3**).

**Table 3:** Effect of physical activity on the prevention of depressive symptoms in menopausal women.

Outcome	Number of studies	SMD (95%CI)	*p*		I^2^
Depressive symptoms	6	−1.23 (−2.21, −0.24)	0.015		94.4%
Sensitivity analysis		−1.23 (−2.21, −0.24)			
Exercise intensity					
Moderate	3	−1.57(−3.71, 0.57)	0.150		96.7%
Low	1	−1.63 (−2.03, −1.23)	<0.001		0.0%
Exercise form					
IE	4	−1.32 (−2.90,0.25)	0.100		96.2%
TE	2	−1.04 (−2.26,0.18)	0.093		89.1%
Intervention duration					
> 12 weeks	3	−2.06 (−3.44, −0.68)	0.003		95.7%
≤ 12 weeks	3	−0.32 (−0.72,0.08)	0.113		0.2%
Supervised					
Yes	4	−1.59 (−2.91, −0.27)	0.018		95.1%
No	2	−0.51 (−1.46, 0.45)	1.299		81.5%
Types of scale					
BDI	1	−3.59 (−4.17, −3.01)	<0.001		0.0%
GDS	2	−0.32 (−1.01, 0.36)	0.359		48.9%
CES-D	1	−1.63 (-2.03, −1.23)	<0.001		0.0%
SDS	1	−0.97 (−1.49, −0.46)	<0.001		0.0%
DASS-21	1	−0.39 (−1.09, 0.31)	0.279		0.0%
Sample size					
≥100	2	−2.60 (−4.51, −0.68)	0.008		96.6
<100	4	−0.53 (−0.98, −0.09)	0.019		48.3
Geographical region					
Europe	1	−3.59 (−4.17, −3.01)	<0.001		0.0
East Asia	4	−0.86 (−1.57, −0.15)	0.018		84.6
Middle East	1	−0.39 (−1.09, 0.31)	0.279		0.0

SMD, standard mean difference; CI, confidence interval; IE, individual exercise; TE, team exercise; BDI, Beck Depression Inventory; GDS, Geriatric Depression Scale; CES-D, Center for Epidemiologic Studies Depression Scale; SDS, Self-Rating Depression Scale.

The regression analyses were conducted on the duration of intervention, exercise form, supervised, and sample size, with results showing a *p*-value >0.05. This suggested that the duration of intervention, exercise form, supervised, and sample size were unlikely to be a source of heterogeneity in the outcome (**Supplementary Table S1**).

### Effect of physical activity on the treatment of depression in menopausal women

**Figure 4:**
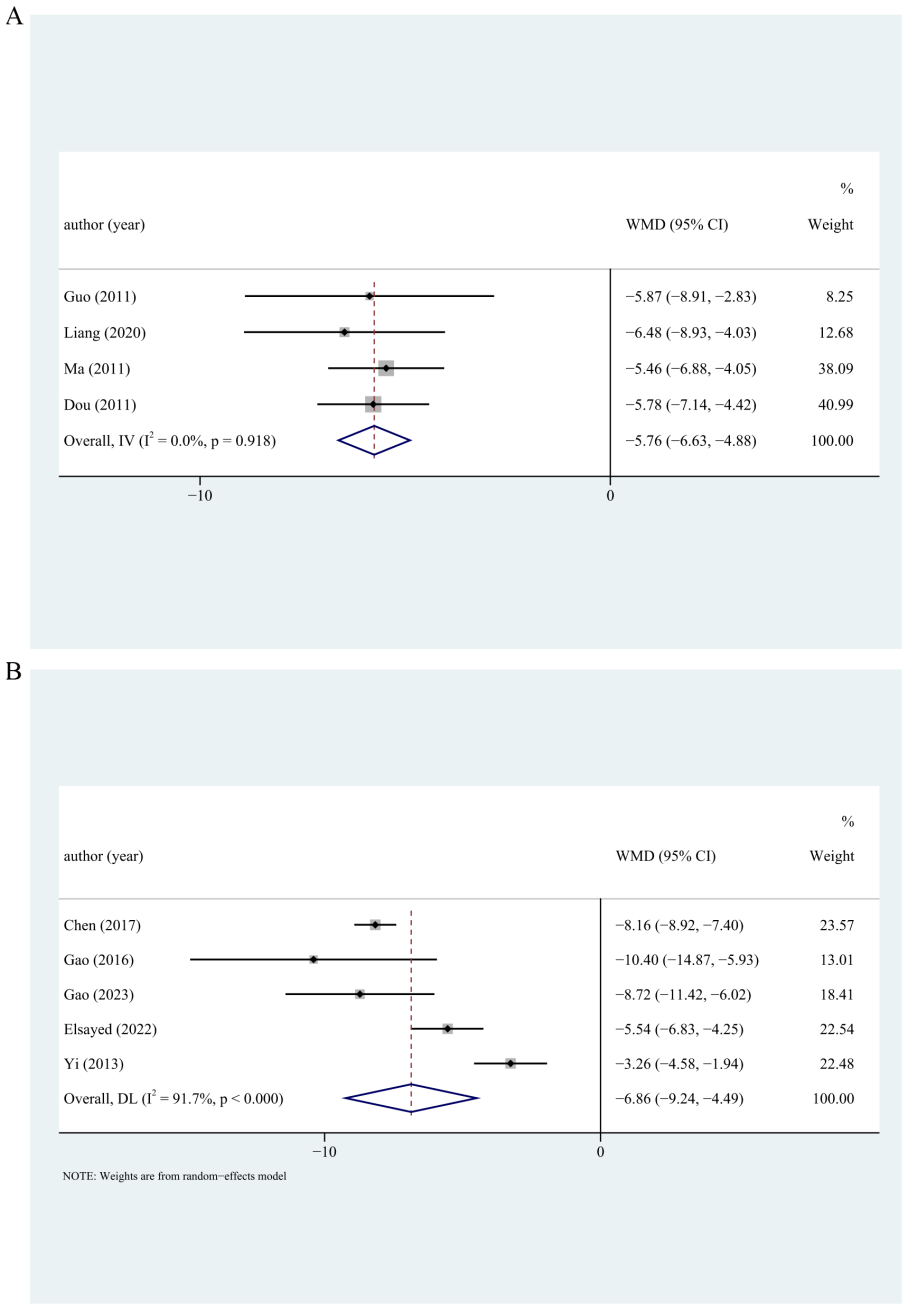
Forest plots of the effects of physical activity on depression assessed by CES-D **(A)** and SDS **(B)**. CES-D, Center for Epidemiologic Studies Depression. Scale; SDS, Self-Rating Depression Scale.

Four studies reported depression assessed by CES-D. The pooled results showed that exercise improved depression in menopausal women (WMD = −5.76; 95% CI, −6.63 to −4.89; I^2^ = 0.0%) (**Figure 4A**). The similar results were found in menopausal women receiving vigorous (WMD = −6.24; 95% CI, −8.15 to −4.33), moderate (WMD = −5.47; 95% CI, −6.88 to −4.05), moderate–vigorous (WMD = −5.78; 95% CI, −7.14 to −4.42) exercise intensity, with exercise >12 weeks (WMD = −5.78; 95% CI, −7.14 to −4.42) or ≤12 weeks (WMD = −5.74; 95% CI, −6.87 to −4.61). Five studies reported depression assessed by SDS. The pooled results showed that depression was alleviated after exercise (WMD = −6.86; 95% CI, −9.24 to −4.49; I^2^ = 91.7%) (**Figure 4B**). Similar results were found in menopausal women with different depression degrees, exercise intensity, intervention duration, exercise form, supervision or not, sample size, and geographical region (**Table 4**). In our analysis, the SMD was also used as the summary effect measure for assessing the impact of physical activity on the treatment of depression in menopausal women. The result showed a significant reduction in depression with physical activity treatment (SMD = −1.45; 95% CI, −1.75 to −1.15) (**Table 4**).

**Table 4:** Effect of physical activity on depression in menopausal women.

Outcome	Number of studies	WMD/SWD (95%CI)	*p*	I^2^
Depression				
CES-D	4	−5.76 (−6.63, −4.88)	<0.001	0.0%
Sensitivity analysis		−5.76 (−6.63, −4.88)		
Exercise intensity				
Vigorous	2	−6.24 (−8.15, −4.33)	<0.001	0.0%
Moderate	1	−5.47 (−6.88, −4.05)	<0.001	0.0%
Moderate–vigorous	1	−5.78 (−7.14, −4.42)	<0.001	0.0%
Intervention duration				
> 12 weeks	1	−5.78 (−7.14, −4.42)	<0.001	0.0%
≤ 12 weeks	3	−5.74 (−6.87, −4.61)	<0.001	0.0%
SDS	5	−6.86 (−9.24, −4.49)	<0.001	91.7%
Sensitivity analysis		−6.86 (−9.24, −4.49)		
Depression degree				
Mild depression	2	−6.91 (−9.47, −4.34)	<0.001	91.5%
Mild to moderate depression	2	−5.86 (−11.20, −0.51)	0.032	92.1%
Exercise intensity				
Moderate	3	−6.64 (−10.28, −3.01)	<0.001	95.2%
Vigorous	1	−10.40 (−14.87, −5.93)	<0.001	0.0%
Low-moderate	1	−5.54 (−6.83, −4.25)	<0.001	0.0%
Intervention duration				
> 12 weeks	2	−5.74 (−10.55, −0.94)	0.019	97.5%
≤ 12 weeks	3	−7.72 (−10.68, −4.77)	<0.001	73.7%
Exercise form				
IE	2	−6.90 (−9.99, −3.82)	<0.001	95.2%
TE	3	−6.95 (−10.97, −2.93)	0.001	77.0%
Supervised				
Yes	4	−6.46 (−9.18, −3.74)	<0.001	93.5%
No	1	−8.72 (−11.42, −6.02)	<0.001	0.0%
Sample size				
≥100	1	−3.26 (−4.58, −1.94)	<0.001	0.0%
<100	4	−7.71 (−9.59, −5.84)	<0.001	78.6%
Geographical region				
East Asia	4	−7.35 (−10.56, −4.13)	<0.001	93.1%
Middle East	1	−5.54 (−6.834, −4.25)	<0.001	0.0%
Overall	11	−1.45 (−1.75, −1.15)	<0.001	76.2%
Sensitivity analysis		−1.45 (−1.75, −1.15)		
Begg’s test		Z=1.56	0.119	
Egger’s test		t=−1.77	0.110	

WMD, weighted mean difference; SMD, standard mean difference; CI, confidence interval; BDI, Beck Depression Inventory; GDS, Geriatric Depression Scale; CES-D, Center for Epidemiologic Studies Depression Scale; SDS, Self-Rating Depression Scale; IE, individual exercise; TE, team exercise.

This meta-analysis further conducted a regression analysis to explore potential factors contributing to heterogeneity. In the regression analysis, the result found that the duration of intervention, exercise form, and supervision did not have a significant impact on the outcomes (*p* > 0.05), indicating that these variables were not sources of heterogeneity (**Supplementary Table S2**).

Abedi et al. (1) reported depression assessed by the BDI and found that the depression level was reduced after 12 weeks of exercise. Gutiérrez et al. (28) reported depression assessed by GDS and found that physical exercise for menopausal women alleviated the depression.

### Sensitivity analysis

The results of the sensitivity analysis showed no significant changes in the overall effect estimates when individual studies were systematically excluded. This consistency across analyses suggests that our findings are robust and not unduly influenced by any single study. The stability of these results supports the reliability of our conclusions.

## Discussion

This meta-analysis explored the effect of physical activity on menopausal women with determined depression or depressive symptoms. The results showed that depressive symptoms of menopausal women were alleviated after exercise. The result was similar in menopausal women receiving low intensity of exercise, team exercise, exercise > 12 weeks, or under supervision. In addition, exercise improved depression in menopausal women. The result was similar regardless of depression degrees, exercise intensity, intervention duration, exercise form, supervision or not, sample size, and geographical region.

Menopause is a natural and inevitable stage in the aging process of women, which is caused by the depletion of ovarian follicles (3). Menopausal women are more susceptible to facing psychological problems under multiple pressures from society, family, work, and life, and some of them may exhibit depressive symptoms or suffer from depression (3, 27). The risk of depressive symptoms or depressive disorders doubles to quadruples during the menopausal transition (3). Exercise can maintain the level of estrogen and progesterone in the body and enhance the functions of the cardiovascular, respiratory, and nervous systems (27). Physical activities affect individuals’ physiology and psychology and are regarded as an effective behavior intervention for depression (37). Existing evidence has shown that physical activity is beneficial for the prevention of depression (38, 39). Kim et al. found in a cohort study that an optimal amount of physical activity reduced the onset of depressive symptoms (38). Li et al. also found that running exercise protected hippocampal astrocytes and decreased the production of new astrocytes, thus preventing the central nervous system from damage and reducing the occurrence of depressive symptoms (39). In this meta-analysis, we found that physical activity alleviated depressive symptoms in menopausal women, indicating that physical activity could be a feasible therapy to prevent depression in menopausal women. A cross-sectional study reported that exercise intensity, duration, and type were closely related to depression (40). Low intensity exercise, such as yoga, has been reported to enhance menopausal women’s ability to control negative emotional experiences and have a positive effect on improving their mental state, thereby improving depressive symptoms (34). This meta-analysis found that low-intensity exercise alleviated the depressive symptoms of menopausal women. Our meta-analysis also showed that depressive symptoms were improved after team exercise, exercise > 12 weeks, or exercise under supervision, which was consistent with the previous studies (21, 31, 34).

Globally, 35.6% of menopausal women were diagnosed with depression (3), and physical activity is a treatment for patients with determined depression (41). The pooled results of this meta-analysis displayed that physical activity improved determined depression in menopausal women. There are several potential mechanisms accounting for this finding. First, brain-derived neurotrophic factor (BDNF) is lower in older adults and patients with depression (42, 43), and a decrease in BDNF is associated with an increased risk of depression (44). Physical activity is found to increase the brain blood flow, thereby increasing the synthesis and release of BDNF (45), indicating that physical activity may reduce the risk of depression by increasing BDNF concentrations. Second, a decrease in serotonin concentration is found to be associated with depression (46), and exercises have been shown to modulate circulating levels of serotonin in patients with depression (47). Third, depression is associated with the activation of innate immune responses and mild systemic inflammation (48, 49). With the decrease in estrogen level, the protective antioxidant benefits and anti-inflammatory effects of estrogen are weakened, and menopausal women are likely to face an increased risk of depression (50). Exercise is found to exert antidepressant effects by regulating oxidative stress (37). Our meta-analysis also displayed that regardless of depression degree, exercise intensity, intervention duration, exercise form, or whether being supervised, physical activity showed a beneficial role in the treatment of depression. These findings suggested that physical activity might be an effective adjuvant therapy to treat depression in menopausal women.

This meta-analysis exclusively incorporates randomized controlled trials (RCTs), thereby enhancing the methodological rigor of the evidence presented. This study also conducts subgroup analyses based on various factors, including the degree of depression, exercise intensity, type of exercise, duration of intervention, presence or absence of supervision, sample size, and geographical region. In addition, a study indicates that commonly used depression scales all demonstrate good internal consistency (51). The factor analysis results of these scales suggest that, despite their differing factor structures, they complement each other in assessing depressive symptoms or depression. However, there are some limitations in this meta-analysis. First, the considerable variation in exercise types among the included studies prevented us from conducting a detailed analysis by specific exercise modalities. This lack of consistency in intervention types may influence the overall outcomes and contribute to the observed heterogeneity. Consequently, the findings may not fully capture the potential differential effects of various exercise forms. Although we attempted to address this through sensitivity and subgroup analyses, the inherent variability remains a factor that should be considered when interpreting the results. This limitation underscores the need for more standardized interventions in future studies to better isolate and understand the effects of specific types of exercise. Second, the number of studies that reported the depressive symptoms of menopausal women is relatively small, which may affect the stability of the results. In addition, many of the studies included had relatively small sample sizes, which may impact the statistical power of our findings and increase the risk of bias. Smaller sample sizes may lead to less reliable estimates, and caution should be taken when interpreting the results. Third, the quality of some of the literature is low, which could influence the reliability and generalizability of the findings. The lower quality of the studies may introduce bias, such as selective reporting, small sample sizes, and lack of blinding, all of which can affect the validity of the results. Future studies with improved methodological rigor are needed to confirm and strengthen the evidence on this topic. Fourth, a key limitation of our study is the focus on leisure-time physical activity, which does not capture the full range of physical activity domains including occupational, transportation, and housework activities. This narrow scope was chosen due to the predominance of leisure-time physical activity in the existing literature on depression or depressive symptoms in menopausal women, the consistency in measurement across studies, and the relevance of leisure-time physical activity to public health interventions. While our meta-analysis provides specific insights into the impact of leisure-time physical activity, it may not be extrapolated to other forms of physical activity. We acknowledge the need for future research to explore the effects of occupational, transportation, and housework physical activities on mental health in menopausal women, offering a more comprehensive view of physical activity’s role in managing depression during this life stage. Fifth, another major limitation of our study is the high heterogeneity among the studies we analyzed, which could affect the trustworthiness of our results. This is a common issue in meta-analyses with various interventions. To address this, we conducted subgroup analyses. We also performed a meta-regression to investigate the sources of heterogeneity. However, some heterogeneity remains unexplained, indicating a need for more standardized interventions in future research to improve study comparability and strengthen our findings. Sixth, the sensitivity analysis, while useful for identifying the stability of our results, may not fully capture all sources of bias or variation within the studies. Therefore, the findings should be interpreted with caution, and further research with more refined methodologies is needed to address these limitations.

## Conclusion

Given the limitations in study quality and methodology, the evidence on the effect of leisure-time physical activity on depressive symptoms in menopausal women is of low to moderate certainty. While physical activity may be a feasible intervention, further high-quality studies are needed to confirm these findings.

## Data Availability

The raw data supporting the conclusions of this article will be made available by the authors, without undue reservation.
