# Supplementary material for: Effect of leisure-time physical activity on depression and depressive symptoms in menopausal women: a systematic review and meta-analysis of randomized controlled trials
Source: Front Psychiatry. 2025 Jan 30;15:1480623. doi: 10.3389/fpsyt.2024.1480623 (PMC11821641; doi:10.3389/fpsyt.2024.1480623)
Supplement: Supplementary file 1 [file Table1.docx]

Supplementary Table 1 A regression analysis of effect of physical activity on the prevention of depressive symptoms in menopausal women

| Variable | Coef | Std.Err | t | P | 95%CI | |
| --- | --- | --- | --- | --- | --- | --- |
| duration of intervention | -.9746823 | 1.145538 | -0.85 | 0.551 | -15.53013 | 13.58076 |
| exercise form | 1.180147 | 0.8169865 | 1.44 | 0.385 | -9.200651 | 11.56094 |
| supervised | -1.141337 | 1.094672 | -1.04 | 0.487 | -15.05046 | 12.76779 |
| Sample size | -1.0706 | 1.407594 | -0.76 | 0.586 | -18.95578 | 16.81458 |

Notes: CI: confidence interval.

Supplementary Table 2 A regression analysis of duration of intervention, exercise form, and supervision on depression in menopausal women

| Variable | Coef | Std.Err | t | *P* | 95%CI | |
| --- | --- | --- | --- | --- | --- | --- |
| Duration of intervention | 4.657764 | 4.787816 | 0.97 | 0.509 | -56.1772 | 65.49273 |
| Exercise form | -4.860001 | 5.39138 | -0.9 | 0.533 | -73.36398 | 63.64398 |
| Supervised | 3.180001 | 5.075533 | 0.63 | 0.644 | -61.31076 | 67.67076 |

Notes: CI: confidence interval.
