# Supplementary material for: Effect of leisure-time physical activity on depression and depressive symptoms in menopausal women: a systematic review and meta-analysis of randomized controlled trials
Source: Front Psychiatry. 2025 Jan 30;15:1480623. doi: 10.3389/fpsyt.2024.1480623 (PMC11821641; doi:10.3389/fpsyt.2024.1480623)
Supplement: Supplementary file 2 [file Table2.docx]

Supplementary File 1 search strategy of PubMed database (Last updated November 13th, 2024)

| #1 | ((((((Depression[MeSH Terms]) OR (Depressive Symptoms[Title/Abstract])) OR (Depressive Symptom[Title/Abstract])) OR (Symptom, Depressive[Title/Abstract])) OR (Emotional Depression[Title/Abstract])) OR (Depression, Emotional[Title/Abstract])) |
| --- | --- |
| #2 | ((((((perimenopause[MeSH Terms]) OR (menopause[MeSH Terms])) OR (menopausal[Title/Abstract])) OR (postmenopause[MeSH Terms])) OR (postmenopausal[Title/Abstract])) OR (climacteric[Title/Abstract])) OR (perimenopausal[Title/Abstract]) |
| #3 | (((((((((((((((((((((((((Exercise[MeSH Terms]) OR (Exercises[Title/Abstract])) OR (Physical Activity[Title/Abstract])) OR (Activities, Physical[Title/Abstract])) OR (Activity, Physical[Title/Abstract])) OR (Physical Activities[Title/Abstract])) OR (Exercise, Physical[Title/Abstract])) OR (Exercises, Physical[Title/Abstract])) OR (Physical Exercise[Title/Abstract])) OR (Physical Exercises[Title/Abstract])) OR (Acute Exercise[Title/Abstract])) OR (Acute Exercises[Title/Abstract])) OR (Exercise, Acute[Title/Abstract])) OR (Exercises, Acute[Title/Abstract])) OR (Exercise, Isometric[Title/Abstract])) OR (Exercises, Isometric[Title/Abstract])) OR (Isometric Exercises[Title/Abstract])) OR (Isometric Exercise[Title/Abstract])) OR (Exercise, Aerobic[Title/Abstract])) OR (Aerobic Exercise[Title/Abstract])) OR (Aerobic Exercises[Title/Abstract])) OR (Exercises, Aerobic[Title/Abstract])) OR (Exercise Training[Title/Abstract])) OR (Exercise Trainings[Title/Abstract])) OR (Training, Exercise[Title/Abstract])) OR (Trainings, Exercise[Title/Abstract]) |
| #4 | ((((((((((((((Aerobic[Title/Abstract]) OR (Jog[Title/Abstract])) OR (Walk[Title/Abstract])) OR (Pilates[Title/Abstract])) OR (Strength training[Title/Abstract])) OR (Stretching[Title/Abstract])) OR (Ambulation[Title/Abstract])) OR (Yoga[Title/Abstract])) OR (Swim[Title/Abstract])) OR (Dance [Title/Abstract])) OR (Dancing[Title/Abstract])) OR (strengthening[Title/Abstract])) OR (warming up[Title/Abstract])) OR (cooling down[Title/Abstract])) |
| #5 | ((((((((((((((((((((((((((Exercise[MeSH Terms]) OR (Exercises[Title/Abstract])) OR (Physical Activity[Title/Abstract])) OR (Activities, Physical[Title/Abstract])) OR (Activity, Physical[Title/Abstract])) OR (Physical Activities[Title/Abstract])) OR (Exercise, Physical[Title/Abstract])) OR (Exercises, Physical[Title/Abstract])) OR (Physical Exercise[Title/Abstract])) OR (Physical Exercises[Title/Abstract])) OR (Acute Exercise[Title/Abstract])) OR (Acute Exercises[Title/Abstract])) OR (Exercise, Acute[Title/Abstract])) OR (Exercises, Acute[Title/Abstract])) OR (Exercise, Isometric[Title/Abstract])) OR (Exercises, Isometric[Title/Abstract])) OR (Isometric Exercises[Title/Abstract])) OR (Isometric Exercise[Title/Abstract])) OR (Exercise, Aerobic[Title/Abstract])) OR (Aerobic Exercise[Title/Abstract])) OR (Aerobic Exercises[Title/Abstract])) OR (Exercises, Aerobic[Title/Abstract])) OR (Exercise Training[Title/Abstract])) OR (Exercise Trainings[Title/Abstract])) OR (Training, Exercise[Title/Abstract])) OR (Trainings, Exercise[Title/Abstract])) OR (((((((((((((((Aerobic[Title/Abstract]) OR (Jog[Title/Abstract])) OR (Walk[Title/Abstract])) OR (Pilates[Title/Abstract])) OR (Strength training[Title/Abstract])) OR (Stretching[Title/Abstract])) OR (Ambulation[Title/Abstract])) OR (Yoga[Title/Abstract])) OR (Swim[Title/Abstract])) OR (Dance [Title/Abstract])) OR (Dancing[Title/Abstract])) OR (strengthening[Title/Abstract])) OR (warming up[Title/Abstract])) OR (cooling down[Title/Abstract]))) |
| #6 | (((((((Depression[MeSH Terms]) OR (Depressive Symptoms[Title/Abstract])) OR (Depressive Symptom[Title/Abstract])) OR (Symptom, Depressive[Title/Abstract])) OR (Emotional Depression[Title/Abstract])) OR (Depression, Emotional[Title/Abstract]))) AND (((((((perimenopause[MeSH Terms]) OR (menopause[MeSH Terms])) OR (menopausal[Title/Abstract])) OR (postmenopause[MeSH Terms])) OR (postmenopausal[Title/Abstract])) OR (climacteric[Title/Abstract])) OR (perimenopausal[Title/Abstract])) |
| #7 | ((((((((Depression[MeSH Terms]) OR (Depressive Symptoms[Title/Abstract])) OR (Depressive Symptom[Title/Abstract])) OR (Symptom, Depressive[Title/Abstract])) OR (Emotional Depression[Title/Abstract])) OR (Depression, Emotional[Title/Abstract]))) AND (((((((perimenopause[MeSH Terms]) OR (menopause[MeSH Terms])) OR (menopausal[Title/Abstract])) OR (postmenopause[MeSH Terms])) OR (postmenopausal[Title/Abstract])) OR (climacteric[Title/Abstract])) OR (perimenopausal[Title/Abstract]))) AND (((((((((((((((((((((((((((Exercise[MeSH Terms]) OR (Exercises[Title/Abstract])) OR (Physical Activity[Title/Abstract])) OR (Activities, Physical[Title/Abstract])) OR (Activity, Physical[Title/Abstract])) OR (Physical Activities[Title/Abstract])) OR (Exercise, Physical[Title/Abstract])) OR (Exercises, Physical[Title/Abstract])) OR (Physical Exercise[Title/Abstract])) OR (Physical Exercises[Title/Abstract])) OR (Acute Exercise[Title/Abstract])) OR (Acute Exercises[Title/Abstract])) OR (Exercise, Acute[Title/Abstract])) OR (Exercises, Acute[Title/Abstract])) OR (Exercise, Isometric[Title/Abstract])) OR (Exercises, Isometric[Title/Abstract])) OR (Isometric Exercises[Title/Abstract])) OR (Isometric Exercise[Title/Abstract])) OR (Exercise, Aerobic[Title/Abstract])) OR (Aerobic Exercise[Title/Abstract])) OR (Aerobic Exercises[Title/Abstract])) OR (Exercises, Aerobic[Title/Abstract])) OR (Exercise Training[Title/Abstract])) OR (Exercise Trainings[Title/Abstract])) OR (Training, Exercise[Title/Abstract])) OR (Trainings, Exercise[Title/Abstract])) OR (((((((((((((((Aerobic[Title/Abstract]) OR (Jog[Title/Abstract])) OR (Walk[Title/Abstract])) OR (Pilates[Title/Abstract])) OR (Strength training[Title/Abstract])) OR (Stretching[Title/Abstract])) OR (Ambulation[Title/Abstract])) OR (Yoga[Title/Abstract])) OR (Swim[Title/Abstract])) OR (Dance [Title/Abstract])) OR (Dancing[Title/Abstract])) OR (strengthening[Title/Abstract])) OR (warming up[Title/Abstract])) OR (cooling down[Title/Abstract])))) |
