# Supplementary material for: Effect of leisure-time physical activity on depression and depressive symptoms in menopausal women: a systematic review and meta-analysis of randomized controlled trials
Source: Front Psychiatry. 2025 Jan 30;15:1480623. doi: 10.3389/fpsyt.2024.1480623 (PMC11821641; doi:10.3389/fpsyt.2024.1480623)
Supplement: Supplementary file 3 [file Table3.docx]

**Author(s):**

**Question:** [intervention] compared to [comparison] for [health problem and/or population]

**Setting:**

**Bibliography:**

| **Certainty assessment** | | | | | | | **№ of patients** | | **Certainty** | **Importance** |
| --- | --- | --- | --- | --- | --- | --- | --- | --- | --- | --- |
| **№ of studies** | **Study design** | **Risk of bias** | **Inconsistency** | **Indirectness** | **Imprecision** | **Other considerations** | **[intervention]** | **[comparison]** |  |  |
| **Treatment-depression(CES-D scale)** | | | | | | | | | | |
| 4 | Randomized trials | Serious^a^ | Not serious | Not serious | Not serious | None | 273 | 177 | ⨁⨁⨁◯ Moderate^a^ | CRITICAL |
| **Treatment-depression(SDS scale)** | | | | | | | | | | |
| 5 | Randomized trials | Serious^b^ | Serious^c^ | Not serious | Not serious | None | 222 | 168 | ⨁⨁◯◯ Low^b,c^ | CRITICAL |
| **Prevention-depression** | | | | | | | | | | |
| 4 | Randomized trials | Serious^b^ | Serious^c^ | Not serious | Not serious | None | 162 | 155 | ⨁⨁◯◯ Low^b,c^ | CRITICAL |

**CI:** confidence interval; **MD:** mean difference; **SMD:** standardized mean difference

#### Explanations

a. No blinding was used

b. The method was used improperly

c. High heterogeneity
